# Can Large Language Models Replace Therapists? Evaluating Performance at Simple Cognitive Behavioral Therapy Tasks

**DOI:** 10.2196/52500

**Published:** 2024-07-30

**Authors:** Nathan Hodson, Simon Williamson

**Affiliations:** 1 Warwick Medical School University of Warwick Coventry United Kingdom

**Keywords:** mental health, psychotherapy, digital therapy, CBT, ChatGPT, cognitive behavioral therapy, cognitive behavioural therapy, LLM, LLMs, language model, language models, NLP, natural language processing, artificial intelligence, performance, chatbot, chatbots, conversational agent, conversational agents

## Abstract

The advent of large language models (LLMs) such as ChatGPT has potential implications for psychological therapies such as cognitive behavioral therapy (CBT). We systematically investigated whether LLMs could recognize an unhelpful thought, examine its validity, and reframe it to a more helpful one. LLMs currently have the potential to offer reasonable suggestions for the identification and reframing of unhelpful thoughts but should not be relied on to lead CBT delivery.

## Introduction

Large language models (LLMs) represent a significant advance in the field of artificial intelligence (AI) and herald a transformational change in the role of computers both personally and professionally. LLMs, such as OpenAI’s ChatGPT and Google’s Bard (later rebranded as Gemini), represent a new form of generative AI. They have linguistic capabilities comparable to humans, and they demonstrate performance similar to specialized models for sentiment analysis and affective computing [1]. Psychiatry and psychology, and talking therapy, in particular, is a field with significant potential impact of LLMs. Demand for therapists greatly outweighs supply, making the question of how new technologies could relieve pressure on mental health systems a pertinent one. Here we report an evaluation of whether existing LLMs can contribute to the delivery of cognitive behavioral therapy (CBT), and their limitations.

CBT is a first-line treatment for common mental health disorders, including anxiety and depression. It involves understanding cognitive biases and challenging those thoughts. Where other modes of psychotherapy rely on the therapist’s individualized interpretation, CBT emphasizes systematic changes in thinking and behavior.

Self-guided, web-based CBT has emerged as a response to the shortage of CBT therapists, and it is increasingly recommended as an accessible alternative [2]. These programs reduce the input of the human therapist to a brief phone call, with patients assigned web-based modules to complete. Although the approach is cost-effective and scalable, it risks making the content of web-based CBT less personalized. Since LLMs can flexibly respond to personal circumstances, they may be well-suited to addressing this.

AI has previously been used to augment CBT by performing peripheral tasks. In a study of chronic pain, AI was used to select the appropriate CBT intervention for patients each week based on the previous week's progress [2]. The digital CBT company Wysa [3] uses AI to select appropriate therapist-authored responses. Mental Health America has built a website using AI to help people identify and reframe cognitive biases as an isolated exercise [4]. However, none of these applications have harnessed the generative capacity of LLMs as therapeutic chatbots to aid patients in reframing unhelpful thoughts.

We aimed to understand whether AI could recognize an unhelpful thought, examine its validity, and reframe it to a more helpful one. This technique, often referred to as “catch it, check it, change it,” requires knowledge of cognitive biases, the linguistic ability to reframe them, and importantly, a degree of comprehension such that the reframing meaningfully addresses the bias [5]. If publicly available LLMs can support “Catch It, Check It, Change It,” then they may have a valuable role in increasing the effectiveness of digital CBT.

## Methods

We explored whether OpenAI’s ChatGPT-4 and Google’s Bard could perform the 3 stages of the “catch it, check it, change it” technique (see Table 1). Two independent CBT therapists currently practising in the UK’s National Health Service aided in assessing the LLMs, rating whether they had completed the tasks satisfactorily. The therapists each wrote their own set of 10 thoughts, ensuring they received different replies from the LLMs. Both ChatGPT-4 and Bard responded to 20 tasks at each stage of the study. The sessions for each therapist occurred on June 2 and 14, 2023.

**Table 1:** Evaluating how large language models (LLMs) perform at the Catch It, Check It, Change It approach.

	CBT^a^ skill	Input to LLM	Task for LLM	Criteria
Stage 1: “Catch it”	“Catch it” means patients can stop and notice that their thought may be distorted. Therapists must be able to illustrate different distortions.	Titles of 10 cognitive biases	Generate a two-sentence vignette for each bias.	Could CBT therapists work out which bias each vignette illustrated?
Stage 2: “Check it”	“Check it” means patients consider whether a thought is helpful, or whether it fits with a cognitive distortion. Therapists must be able to explain which distortion a thought fits into.	Therapist-written thoughts illustrating 10 cognitive distortions, each in the language of a patient. Each therapist produced an independent list of thoughts with no discussion.	Identify which cognitive bias each vignette represents.	Did LLMs identify the same biases?
Stage 3: “Change it”	“Change it” means patients can reframe their thoughts. Therapists should be able to suggest reframing of thoughts that patients may consider.	Therapist-written thoughts illustrating 10 cognitive biases as above	Reframe the thought to overcome the bias.	Did therapists think the new thought addressed the bias?

^a^CBT: cognitive behavioral therapy.

## Results

Table 2 shows LLM performance over the 3 tasks. Both models demonstrated varying levels of proficiency across tasks and therapists. Overall, ChatGPT-4 scored 44/60 and Bard scored 42/60. Both performed similarly at generating vignettes, which clearly illustrated a cognitive bias (Stage 1: ChatGPT 13/20, Bard 13/20), whereas ChatGPT-4 performed better at identifying cognitive biases (Stage 2: ChatGPT 15/20, Bard 10/20). The LLMs performed superiorly at reframing unhelpful thoughts, with Bard achieving a near-perfect score (Stage 3: ChatGPT 16/20, Bard 19/20).

Frequently, the LLMs were only marginally incorrect. Specifically, Bard often mentioned cognitive biases outside of the 10 provided, using alternative labels that nonetheless described the bias plausibly. This may reflect an inherent limitation of CBT terminology, rather than poor model performance. Indeed, this limitation appeared to extend to therapists, who only demonstrated moderate inter-rater reliability in labeling LLM-generated vignettes (Cohen κ=0.44). However, at stage 3, therapist 2 noted several instances where the LLM “missed the point*”* and, while technically improving the original thought, did not reframe it in a way that demonstrated understanding of the underlying cognitive bias. Prompts given to these LLMs and examples of errors noted in the outputs are presented in Multimedia Appendix 1.

**Table 2:** Number of tasks completed correctly at each stage.

Evaluation stage	Bard				ChatGPT-4		
	Therapist 1 (out of 10)	Therapist 2 (out of 10)	Total	Therapist 1 (out of 10)		Therapist 2 (out of 10)	Total
Stage 1: Catch it (How many LLM-generated vignettes were correctly identified by a therapist?)	7	6	13	8		5	13
Stage 2: Check it (How many therapist-generated vignettes were correctly identified by the LLM?)	7	3	10	7		8	15
Stage 3: Change it (How many LLM-reformulated vignettes were considered improvements by a therapist?)	10	9	19	10		6	16

## Discussion

Our study findings suggest that LLMs should not yet be relied on to lead CBT delivery, although LLMs show clear potential as assistants capable of offering reasonable suggestions for the identification and reframing of unhelpful thoughts.

LLMs are far from replacing CBT therapists, but they perform well in some isolated tasks (eg, Bard for reframing), so it is worthwhile exploring limited yet innovative ways to use AI to improve patient experience and outcomes. We suggest CBT therapists equip patients with a working knowledge of cognitive biases, but therapists could also advise patients to consider using LLMs to gather suggestions on reframing unhelpful thoughts beyond sessions.

## Appendix

Multimedia Appendix 1 Prompts provided to the large language models Bard and ChatGPT-4 and examples of errors noted in the outputs.
