# Supplementary material for: Can Large Language Models Replace Therapists? Evaluating Performance at Simple Cognitive Behavioral Therapy Tasks
Source: JMIR AI. 2024 Jul 30;3:e52500. doi: 10.2196/52500 (PMC11322688; doi:10.2196/52500)
Supplement: Multimedia Appendix 1 [file ai_v3i1e52500_app1.docx]

**Table S1.** Prompts given to the large language models Bard and ChatGPT-4.

| Initialization | Below is a list of cognitive distortions:   1. All or nothing thinking: You look at things in absolute, black and white categories. 2. Overgeneralisation: You view a negative event as a never-ending pattern of defeat. 3. Mental Filter: You dwell on the negatives and ignore the positives. 4. Discounting the positives: You insist that your accomplishments or positive qualities “don’t count”. 5. Jumping to conclusions: (A) Mind reading – you assume that people are reacting negatively to you when there’s no definite evidence for this; (B) Fortune Telling – you arbitrarily predict things will turn out badly. 6. Magnification or Minimization: You blow things way out of proportion or you shrink their importance inappropriately. 7. Emotional Reasoning: You reason from how you feel: “I feel like an idiot, so I really must be one”. Or “I don’t feel like doing this, so I’ll put it off”. 8. Should Statements: You criticize yourself or other people with “Shoulds” or “Shouldn’ts,” “Musts,” “Oughts,” “Have tos” are similar offenders. 9. Labeling: You identify with your shortcomings. Instead of saying “I made a mistake”, you tell yourself “I’m a jerk”, or “a fool”, or “a loser”. 10. Personalization and Blame: You blame yourself for something you weren’t entirely responsible for. |
| --- | --- |
| Stage 1: *Catch it* | *Please generate a two-sentence vignette for each cognitive distortion. Write it in first person, as if you were a child, and as if you have no insight into the cognitive distortion that is being demonstrated. Each vignette should be longer than 15 words.*  *For magnification or minimization, you only need to give an example of one or the other.*  *For Jumping to conclusions, you only need to give an example of Mind Reading or Fortune telling, not both.* |
| Stage 2: *Check it* | *Please identify, of the 10 cognitive distortions, which is demonstrated in the following vignette:*  ENTER THERAPIST VIGNETTE HERE  (Repeat x10 for each therapist-generated vignette) |
| Stage 3: *Change it* | *Please reformulate the vignette given to a healthier form of the same thought.*  (Repeat x10 for each therapist-generated vignette) |

**Table S2.** Examples of errors made by the large language models Bard and ChatGPT-4.

| Evaluation stage | Example |
| --- | --- |
| Stage 1: *Catch it* | LLM-generated vignette for “All or Nothing” thinking:   - “I got one question wrong on my test, so I must be a failure.”   Therapist-assigned label:   - Mental filter |
| Stage 2: *Check it* | Therapist-generated vignette for “All or Nothing” thinking:   - “When I look at my life it’s all rubbish. Nothing good has ever happened to me.”   LLM-assigned label:   - Overgeneralisation (of note, the LLMs often gave rationale for their labels unprompted, as in this example: “The individual is viewing their entire life as negative and not acknowledging any positive events, implying a never-ending pattern of defeat.”) |
| Stage 3: *Change it* | Therapist-generated vignette for “Labeling”:   - “Dad said everything he does is for us kids, I don’t do everything for others so that must mean I’m really selfish.”     Reformulated by LLM, and considered by the therapist not to be an improvement:   - “Dad is doing his best for us, and I can try to be considerate in my own way.” |
